# The prevalence of intestinal parasites, undernutrition and their associated risk factors among school‐age children in Sekota Town, Northeast Ethiopia: A community‐based cross‐sectional study

**DOI:** 10.1002/hsr2.1137

**Published:** 2023-02-26

**Authors:** Habtu Debash, Megbaru Alemu, Habtye Bisetegn

**Affiliations:** ^1^ Deparment of Medical Laboratory Sciences, College of Medicine and Health Sciences Wollo University Dessie Ethiopia; ^2^ Department of Medical Laboratory Sciences, College of Medicine and Health Sciences Bahirdar University Bahirdar Ethiopia

**Keywords:** ethiopia, intestinal parasites, school age children, Sekota Town, undernutrition

## Abstract

**Background and Aims:**

In developing countries, intestinal parasitic infections and malnutrition are among the most serious health issues affecting school‐aged children. They have synergetic consequences. This study aimed to determine the prevalence of intestinal parasites, undernutrition, and their associated risk factors among school‐age children.

**Methods:**

A community‐based cross‐sectional study was conducted from April to June 2021 among school‐age children in Sekota Town, Northeast Ethiopia. Households were selected using a systematic random sampling technique. Risk factor variables were collected using pretested questionnaires. Stool samples were collected from study participants and examined using a wet mount, formol‐ether concentration, and modified acid‐fast techniques. The height and weight of children were also measured using a meter and a standard calibrated balance, respectively. Data were analyzed using SPSS version 26.0 statistical software.

**Results:**

The overall prevalence of intestinal parasites among school‐age children was 44.3% (178/402). About seven species of intestinal parasites were identified. The predominant parasite identified was *E. histolytica/dispar* (11.2%), followed by *H. nana* (9.2%) and *G. lamblia* (6.7%). The well as a source of drinking water (adjusted odds ratio [AOR] = 7.93; 95% confidence interval [CI]: 4.38–14.36), habit of open‐field defecation (AOR = 7.02; 95% CI: 13.05–12.06), and being undernourished (AOR = 5.67; 95% CI: 2.98–10.79) were independent predictors of intestinal parasitic infections. On the other hand, the overall prevalence of undernutrition was 46.3%. Undernutrition was significantly more likely in children with a dietary diversity score (DDS) of 3 (AOR = 3.73, 95% CI: 2.37–5.88), meal frequency of no more than three times per day (AOR = 2.00, 95% CI: 1.71–2.98), intestinal parasite infection (AOR = 5.25, 95% CI: 3.24–8.52), and no access to school‐based feeding (AOR = 3.52, 95% CI: 2.17–7.96).

**Conclusion:**

The prevalence of intestinal parasitic infections and undernutrition was high among school‐age children in Sekota Town. The results imply the need for strengthening integrated strategies for the reduction of intestinal parasitic infections and undernutrition.

## 1 INTRODUCTION

Despite being the most preventable and treatable disease, intestinal parasite infections are a severe public health problem in developing countries. Over 267 million preschool‐aged children (PSAC) and 568 million school‐aged children (SAC) worldwide live in locations where intestinal parasites are endemic.^1^ Protozoans and helminths are two types of intestinal parasites. *Ascaris lumbricoides*, *Trichuris trichiuria*, and hookworm are the most common helminthic parasites, affecting around one‐sixth of the world's population.^2^, ^3^ In addition, protozoan parasites, including *Giardia lamblia*, *Entamoeba histolytica*, and cryptosporidium infections, are particularly frequent in underdeveloped countries like Ethiopia and are the leading cause of intestinal morbidity in children.^4^ Intestinal parasitic diseases are typically spread through unsanitary practices such as ingesting ova or cysts from unwashed hands and fingernails, eating and drinking contaminated food and water, and skin penetration by larval stage in unsanitary surroundings.^5^, ^6^

School‐age children have a less developed immune system, poor personal hygiene, and the habit of playing on polluted dirt. Therefore, they are at a higher risk of developing intestinal parasite infections.^7^ Intestinal parasites continue to limit human production by causing abdominal aches, anemia, diarrhea, delayed growth, undernutrition, decreased physical activity, and impaired cognitive development in young children.^8^, ^9^ Ethiopia's Federal Ministry of Health (FMOH) has implemented health education, regular deworming, and clean water, sanitation, and hygiene (WASH) initiatives^10^ to manage and prevent intestinal parasites, with a focus on SAC because they are more vulnerable to infection.^1^, ^11^

There are studies on intestinal parasites among school‐age children in different areas of Ethiopia.^12^, ^13^, ^14^, ^15^ However, the major limitation of the studies was determining intestinal parasite prevalence by examination of a single stool specimen. In addition, opportunistic infections were not evaluated. These numbers may underestimate the prevalence of intestinal parasites. To overcome this limitation, two stool samples from each study participant were requested to be submitted for evaluation on 2 consecutive days. On each day, the samples were examined using a wet mount, formol‐ether concentration, and modified acid‐fast techniques.

According to a report by the World Health Organization (WHO), 178 million children were undernourished, with 20 million of them suffering from the most severe type of malnutrition.^16^ Stunting is prevalent in Sub‐Saharan Africa, with a proportion of SAC impacted as high as 19.0%–26.0% in Ethiopia.^17^ Despite the fact that Ethiopia has seen a steady and substantial decrease in stunting over the last decade, levels remain high, and stark geographical inequities exist. Ethiopia's government declared on July 15, 2015, that it would abolish child malnutrition by 2030, reaffirming its commitment to nutrition as a cornerstone for economic development. So the country established the Seqota Declaration to attain this purpose.^18^

Both intestinal parasitic diseases and malnutrition are common in underdeveloped areas.^9^, ^19^ Intestinal parasitic infections can cause malnutrition in children by consuming nutrients and damaging the intestinal mucosa, resulting in reduced digestion and nutritional absorption. On the other hand, malnutrition may be a risk factor for intestinal parasite infections.^20^, ^21^ School‐age children's generally poor health status is being exacerbated by the synergetic effect of these illnesses, which are regarded as top global health challenges.^19^, ^22^, ^23^ End epidemics of water‐borne diseases and other communicable diseases by 2030, as stated in Ethiopia's Sustainable Development Goal 3. Ethiopia also pledged to reduce noncommunicable disease‐related premature mortality by one‐third through prevention and treatment, as well as promote mental health and well‐being.^24^ These types of studies are critical for evaluating programs in various parts of the country.

Furthermore, there is no information available on the nutritional status and intestinal parasitic infections of children over the age of five, particularly in the study area. So this study was conducted to fill these gaps. This study will increase the intersectoral collaboration of decision‐makers in the areas of health and education. The findings of this study will be useful to the Sekota Declaration program, which has been working on stunting, as well as other concerned bodies. Therefore, this study aimed to assess the prevalence of intestinal parasites, undernutrition, and their associated risk factors among SAC in Sekota Town, Northeast Ethiopia.

## 2 MATERIALS AND METHODS

### 2.1 Study area

This study was conducted in Sekota Town, Waghemra Zone, Amhara Regional State, Northeast Ethiopia. Sekota town is found 720 km from Addis Ababa, which is the capital city of Ethiopia. The town is situated at 2266 m above sea level, and its average annual temperature and rainfall are 29°C and 786 mm, respectively. The town is divided into four kebeles (the smallest administrative units next to the district in Ethiopia). Based on the 2021 city administration report, it is home to about 41,696 people and 10,055 households. The town also has one hospital, one health center, and four health posts.

### 2.2 Study design and period

A community‐based cross‐sectional study was conducted from April to June 2021.

### 2.3 Eligibility criteria

Children who are 6–14 years old, who have lived in the town for at least 6 months, and who are willing to participate in the study were included. Children who are taking antiparasitic medication or nutritional supplements and whose guardian is unwilling to give written consent were excluded.

### 2.4 Sample size and sampling methods

The sample size for this study was determined using a single population proportion formula.

$$\text{Study participants}(\text{households})\;:n=\underline{Z{\left(\alpha /2\right)}^{2}\;P\left(1-P\right)/d^{2}}\underline{{\left(1.96\right)}^{2}\;\times \;0.5\left(1–0.5\right)/\left(0.05\right)2}\;=\;384\;\text{and an on}-\text{response rate of}\;10\%=38$$

where *n* = sample size, *z* = 95% statistic for a level of confidence (1.96), *p* = previous prevalence, and *d* = margin of error. Given the 50% prevalence, there have been no previous studies on intestinal parasites and nutritional status in SAC households. Finally, a total of 422 children were included in this study.

### 2.5 Sampling technique and procedures

**Figure 1:**
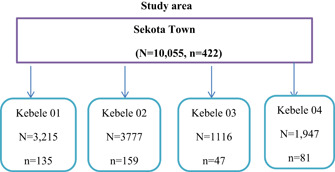
Sampling procedure for a study conducted on intestinal parasites and undernutrition among school‐age children in Sekota Town, Northeast Ethiopia, from April to June 2021.

Using data from the city administration as a sampling frame, stratified sampling was used to allocate the number of households to four kebeles based on their number of households. Based on the total number of households, study participants were proportionally selected from each kebele. The number of households assigned to each kebele was then determined using a systematic random sampling technique for every 24 households. Finally, one school‐age child in a household was selected using the lottery method of random sampling (Figure 1).

### 2.6 Data collection

#### 2.6.1 Socio‐demographic data

A structured, pretested Amharic‐version questionnaire was used to collect socio‐demographic data and other variables through face‐to‐face interview. A pilot test was done in Asketema town with 5% of the sample size before the actual data collection.

#### 2.6.2 Stool sample collection and processing

Approximately 2 g of stool were collected from each study participant using a wide‐mouthed, leak‐proof, and clean stool cup. Because intestinal parasites are shed intermittently, study participants were requested to submit two stool samples for evaluation on 2 consecutive days. Stool samples were labeled with a unique code and transported to the Tefera Hailu Memorial Hospital Laboratory immediately after collection. For the direct saline method, an applicator stick was used to mix about 50 mg of feces with one or two drops of normal saline placed on a clean slide. A cover slip was used to create a thin, uniform suspension. The entire film was screened systematically for the presence of parasites. The remaining samples were then preserved using a modified acid‐fast technique and 10% formalin for formol‐ether concentration.

In addition, using an applicator stick, about 1 g of feces was placed in a clean 15‐mL conical centrifuge tube containing 7 mL of formalin‐saline for the formol‐ether concentration technique. The resulting suspension was filtered through a sieve into another conical centrifuge tube. The debris trapped in the sieve was discarded. After adding 3 mL of diethyl ether to the formalin solution, the contents were centrifuged at 3200 rpm for 3 min. The supernatant was discarded, and the tube was returned to its rack. A smear of the sediment was made on a clean glass slide and covered with a cover slip. Then, the entire area under the coverslip was systematically examined using ×10 and ×40 objective lenses. Furthermore, in the case of modified acid‐fast staining, the fecal smear is methanol‐fixed (1 min) and stained with carbol fuchsin for 5 min. Then it is rinsed with 50% ethanol followed by tap water. It is then decolorized with 1% sulfuric acid for 2 min, washed with tap water, and counter‐stained with alkaline methylene blue for 1 min. Finally, examine the oocyst of intestinal coccidian parasites under a light microscope using a 100‐fold magnification.

#### 2.6.3 Anthropometric measurements

The weight and height of each child were measured by clinical nurses using a digital weighing scale that measured to the nearest 0.1 kg and a vertical meter that measured to the nearest 0.1 cm, respectively. The child's shoes, jacket, and hair clips were removed before measurement, and the children were positioned with their feet together flat on the ground, heels touching the back plate of the measuring instrument, legs straight, buttocks against the backboard, scapula against the backboard, and arms loosely at their sides. To reduce subjectivity error, study participants' weight and height were measured twice by independent nurses, and the mean measurement value was recorded. The WHO AnthroPlus software was used to calculate *Z*‐score of height for age (HAZ) and body mass index for age *Z*‐score (BAZ). Children with *Z*‐score < −2 SD were classified as underweight (WHZ < − 2 SD), stunted (HAZ < − 2 SD) and wasted (BAZ < − 2 SD).^25^

#### 2.6.4 Dietary diversity score

Dietary consumption 24 h before the survey was assessed using questionnaires developed based on Food and Agriculture Organization (FAO) guidelines.^26^ Consumed foods were classified into 10 food groups.^27^ Consumption from each food group was reported, and one point was given if the food was consumed at least once in the last 24 h and zero points if it was not consumed within that period. Therefore, the dietary diversity score (DDS) ranged from 0 to 10, and children with a DDS of ≤3 were classified as having poor diversity, whereas children with a DDS of 4–6 and >6 were classified as having middle and high diversity, respectively.^28^

#### 2.6.5 Quality control

Data collectors and supervisors have been trained in the methods of data collection. The quality of the questionnaires was assessed by pretesting before data collection. On‐the‐spot checking of the completeness of the data was done. All materials, equipment, and procedures were adequately controlled. The stool specimen examination was done following standard laboratory procedures. Negative and positive control slides were used to check the functionality of the microscope as well as the accuracy of the laboratory professional engaged in conducting the study. All slides were examined twice for confirmation of the result.

#### 2.6.6 Data processing and analysis

Data were checked for completeness and entered in SPSS version 26 for analysis. The data were summarized as frequencies, prevalence, and means using descriptive statistics. Bivariable and multivariable logistic regressions were used to identify independent predictors of intestinal parasitic infections and malnutrition. Explanatory variables with a *p* < 0.25 in to the bivariable analysis were entered in to multivariable analysis. Adjusted odds ratios and the corresponding two‐sided 95% confidence intervals (CIs) were used to show the strength of the association. A *p* < 0.05 was considered statistically significant.

#### 2.6.7 Ethical approval and consent to participate

Ethical clearance was obtained from the Ethical Review Committee of the Wollo University College of Medicine and Health Sciences. Permission was obtained from the Sekota Town Health Office, the kebeles, and each household head. The aim of the study was explained to the study participants and their parents or guardians. “Written informed consent was obtained from the parents or guardians of the children.” The confidentiality of the data was maintained. Children who were infected with the intestinal parasite(s) were treated according to the national guidelines, and those with undernutrition were referred to Tefera Hailu Memorial Hospital for further investigation. All methods were carried out in accordance with the Declaration of Helsinki guidelines and regulations.

## 3 RESULTS

### 3.1 Socio‐demographic characteristics

From a total of 422 school‐age children, 402 participated in this study, with a response rate of 95.3%. The mean (±SD) age of study participants was 9.5 (±2.48) years old, with a range of 6 to 14 years. Two hundred two (50.2%) of the study participants were female, and 151/402 (37.6%) of the study participants were from 02 Kebele. About 289/402 (71.9%) of the participants were in grades 1‐4, and more than half of the study participants have a family income of >2500 Ethiopian Birr (Table 1).

**Table 1:** Socio‐demographic characteristics of the study participants in Sekota Town, Northeast Ethiopia, from April to June, 2021.

Variables	Categories	Frequency (*n*)	Percentage (%)
Age groups (years)	6–9	216	53.7
	10–14	186	46.3
Sex	Male	200	49.8
	Female	202	50.2
Kebele	01	130	32.3
	02	151	37.6
	03	43	10.7
	04	78	19.4
Level of grade	1–4	289	71.9
	5–8	113	28.1
Family size	<=4	191	47.5
	>4	211	52.5
Family income per month	≤2500 Et Birr	181	45.0
	>2500 Et Birr	221	55.0
Literacy rate of mother	Secondary school & above	142	35.3
	Primary school	132	32.8
	Illiterate	128	31.8
Literacy rate of the father	Secondary school & above	157	39.1
	Primary school	153	38.1
	Illiterate	92	22.9

### 3.2 Prevalence of intestinal parasitic infections and associated factors

The overall prevalence of intestinal parasites among SAC was 178/402 (44.3%) (95% confidence interval [CI]: 38.6%–49.0%). About 164/402 (40.8%) of children were infected by a single parasite, while 14/402 (3.5%) of them were infected by more than one intestinal parasite at a time. The prevalence of protozoan and helminthic infections was 106/402 (26.4%) and 73/402 (18.2%), respectively. In this study, seven intestinal parasite species were identified. The most predominant parasite identified was *E. histolytica/dispar* 52/402 (11.9%), followed by *H. nana* 38/402 (9.5%) and *G. lamblia* 34/402 (8.5%) (Table 2).

**Table 2:** Prevalence of intestinal parasitic infection among school‐age children in Sekota Town, Northeast Ethiopia, from April to June 2021.

Species of parasites	Number (*n* = 402)	Percent (%)
Single infections		
*Entamoeba histolytica/dispar*	45	11.2
*Hymenolepis nana*	37	9.2
*Giardia lamblia*	27	6.7
*Trichuris trichiuria*	19	4.7
*Cryptosporidium* species	19	4.7
*Enterobius vermicularis*	16	4.0
*Isospora belli*	1	0.2
Total	164	40.8
Double infections		
*Entamoeba histolytica/dispar & Cryptosporidium species*	7	1.7
*Giardia lamblia & Cryptosporidium sppecies*	6	1.5
*Giardia lamblia & Hymenolepis nana*	1	0.2
Total	14	3.5
Overall	178	44.3

Children who drank water from the well had a significantly higher risk of acquiring intestinal parasitic infections (adjusted odds ratio [AOR] = 7.93; 95% CI: 4.383–14.362) as compared to children who drank pipe water. Similarly, children who had the habit of open‐field defecation (AOR = 7.02; 95% CI: 13.05–12.06) and were undernourished (AOR = 5.67; 95% CI: 2.98–10.79) were also independent predictors of intestinal parasitic infections (Table 3).

**Table 3:** Binary logistic regression analysis of factors associated with intestinal parasitic infections among school‐age children in Sekota Town, Northern Ethiopia, from April to June 2021.

Variables	Categories	IPs		Crude OR (95% CI) *p* value	Adjusted OR (95% CI) *p* value
		Pos	Neg		
Literacy rate of mother	≥Secondary school	47	95	1	
	Primary school	59	73	1.591 (0.975–2.596) 0.063	2.262 (0.180–14.335) 0.085
	Illiterate	72	56	2.599 (1.586–4.259) <0.001	2.519 (0.942–14.758) 0.074
Eating raw fruits and vegetables	Yes	98	65	2.997 (1.983–4.528) <0.001	2.064 (0.372–11.439) 0.407
	No	80	159	1	
Water source of drinking	Pipe	80	182	1	
	Well	98	42	5.308 (3.395–8.300) <0.001	7.934 (4.383–14.362) <0.001
Open field defecation	Yes	101	62	3.427 (2.259–5.199) 0.002	7.024 (3.054–12.059) 0.032
	No	77	162	1	
Hand washing after toilet/defecation	Yes	102	172	1	
	No	76	52	2.465 (1.604–3.787) <0.001	1.646 (0.901–10.989) 0.061
Fingernail trim	Yes	86	153	1	
	No	92	71	2.305 (1.535–9.463) 0.031	1.287 (0.5.272‐14.166) 0.071
Soil eating habit	Yes	62	62	1.397 (0.913–2.136) 0.124	
	No	116	162	1	
Presence of domestic animals	Yes	87	61	2.555 (1.686–3.872) <0.001	1.460 (0.728–2.928) 0.286
	No	91	163	1	
Nutritional status	Undernourished	122	64	5.446 (3.546–8.365) <0.001	5.668 (2.978–10.785) <0.001
	Normal	56	160	1	

Abbreviations: CI, confidence interval; OR, odds ratio.

### 3.3 Nutritional status and associated factors

The overall prevalence of undernutrition among SAC was 186/402 (46.3%; 10.2% wasted, 21.6% stunted, and 29.9% underweight). Among undernourished students, 62/186 (33.3%) tested positive for two or more forms of undernutrition at a time (Table 4).

**Table 4:** The prevalence of malnutrition among school‐age children in Sekota Town, Northeast Ethiopia, from April to June 2021.

	**Categories**		**Frequency (*n*)**	**Percentage (%)**
Nutritional status	Malnutrition	Wasted	41	10.2
		Stunted	87	21.6
		Underweight	120	29.9
		≥Forms of undernutrition	62	15.4
		Overall	186	46.3
	Normal		216	53.7

A binary logistic regression model revealed that study participants with a dietary diversity score (DDS) of 3 (AOR = 3.73, 95% CI: 2.37–5.88), meal frequency of at least three times per day (AOR = 2.00, 95% CI: 1.7–2.98), intestinal parasite infection (AOR = 5.25, 95% CI: 3.24–8.52), and no access to school‐based feeding (AOR = 3.52, 95% CI: 2.17–7.96) were strongly associated with under‐nutrition (Table 5).

**Table 5:** Binary logistic regression analysis of potential risk factors associated with under nutrition among school age children in Sekota Town, Nourtheast Ethiopia, from April to June 2021.

Risk factors	Categories	Under nutrition		Crude OR (CI95%) *p*‐value	Adjusted OR (CI 95%) *p*‐value
		Yes	No		
Mother education	≥Secondary school	58	84	1	1
	Primary school	58	74	1.540 (1.044–3.511) 0.084	1.248 (0.717–2.174) 0.433
	Illiterate	70	58	1.748 (1.079–2.833) 0.023	1.302 (0.744–2.277) 0.355
Meal frequency	≤3 times a day	72	51	2.043 (1.328–3.144) <0.001	2.001 (1.709–2.983) 0.015
	>3 times/day	114	165	1	1
Dietary diversity score (DDS)	≤3	126	80	3.570 (2.362–5.397) <0.001	3.727 (2.365–5.875) <0.001
	>3	60	136	1	1
Access to school‐based feeding	Yes	44	125	1	
	No	142	91	4.433 (2.876–6.834) <0.001	3.524 (2.172–7.958) 0.016
Intestinal parasitic infection	Infected	122	56	5.446 (3.546–8.365) <0.001	5.253 (3.238–8.521) <0.001
	Not infected	64	160	1	1

## 4 DISCUSSION

Intestinal parasites are a common public health concern in developing countries, with SAC being at higher risk.^29^ In this study, the overall prevalence of intestinal parasites among SAC in Sekota town was 44.3% (95% CI: 38.6%–49.0%). This finding was higher than reports from Sekota town (30.0%),^30^ Harbu town (21.5%),^12^ Sebaya, northern Ethiopia (29.9%),^13^ Kenya (17.3%),^31^ Nepal (33%),^32^ and Western Iran (9.8%).^33^ This discrepancy might be due to differences in drinking water sources, environmental sanitation, and living conditions among the participants. Furthermore, double samples were examined in this study using a wet mount, concentration, and modified acid‐fast techniques. So examining multiple samples using different methods increases the probability of intestinal parasite detection.^34^

The result of this study was comparable with the number of studies reported in Ethiopia, such as Maksegnit (40.4%), Merawi Town (42.9%), Arbaminch (46.5%),^14^, ^15^, ^35^ the pooled prevalence in Ethiopia (46.09%),^36^ and Eritrea (45.3%).^23^ In contrast, the current study prevalence was lower in comparison with studies done in various areas of the country, such as Mecha district (61.7%), Bahir Dar (52.4%),^9^, ^19^ and in other countries like Sudan (87.2%)^37^ and Argentina (78.1%).^22^ This might be due to the differences in the implementation of health education, regular deworming, and clean water, sanitation, and hygiene (WASH) strategies to manage and prevent intestinal parasites. Furthermore, the higher prevalence reported in Argentina might be due to difference in laboratory methods. Serial fecal samples and anal swab methods were used to increase the detection of intestinal parasites.

Seven species of intestinal parasites were identified. The most common intestinal parasitic infections detected in this study were *E. histolytica/dispar* (11.2%), *H. nana* (9.2%), and *G. lamblia* (6.7%). It was higher compared to other studies conducted in Harbu Town^12^ and Eritrea.^23^ *Entameoba histolytica* causes amoebiasis, a potentially severe and life‐threatening disease and the second most common cause of death from parasitic diseases after malaria. Cryptosporidium species and *G. lamblia* are also nowadays, a major cause of diarrhea, especially in children.^38^, ^39^ The predominance of *E. histolytica/dispar* and *G. lamblia* infection might be associated with poor hygiene, poverty, lack of access to portable water, and a hot, humid tropical climate. Furthermore, their infective stage (the cyst) can withstand a standard level of chlorine treatment in drinking water.^40^ Hence, an unimproved source of drinking water might increase the risk of intestinal protozoan infections among SAC in the study area.

The prevalence rate for *H. nana* was 9.2%, which was consistent with earlier research.^19^, ^23^ This parasite can be transmitted from one child to the next by contaminated hands (external autoinfection) or reverse peristalsis. *T. trichiura* was also the only soil‐transmitted helminthic parasite found in this study (4.7%). It causes different complications like enteropathy as a result ofchronic inflammation, which results in a leaky gut and poor nutrient absorption.^41^, ^42^ Environmental interventions, such as water, sanitation, and hygiene should be considered when designing nutritional interventions.

Intestinal parasites were eight times more likely to infect children who drank well water. This finding was consistent with other studies, which found that using a well as a source of drinking water increased the likelihood of protozoan infection.^23^, ^43^ This could be because human or animal waste can enter the water through various channels if they are not functioning properly. So children can acquire the infection, especially from water‐born intestinal protozoan infections like *G. lamblia* and Cryptosporidium species.

The habit of defecating in the open field was found to be an independent predictor of intestinal parasite infections. Similarly, studies have linked open‐field defication to the spread of intestinal parasite diseases.^44^ Because helminth ova and intestinal protozoan cyst stages can survive for a long time. As a result, children can become infected through a variety of routes. In addition, undernutrition has also been linked to parasitic infections in this study. A study conducted in Bahir Dar, Ethiopia^19^ found a similar correlation. Children are more vulnerable to parasitic infection because malnutrition reduces immune responses.^45^ In this study, 33.3% (122/402) of SAC had intestinal parasitic infections and were undernourished.

On the other hand, undernutrition is one of the most serious health issues facing SAC in poor countries, like Ethiopia, and it can have a negative impact on their physical and mental development.^20^, ^46^, ^47^ In the studied area, 46.3% (95% CI: 41.3%–50.5%) of SAC suffer from malnutrition. This result was consistent with studies from Bahirdar City^19^ and Nigeria.^48^ However, the prevalence of undernutrition in this study was higher than in previously conducted studies among SAC in Jimma Town,^44^ Mecha District,^47^ national figures,^17^ and in Arigentina.^22^ This might be due to differences in the sociocultural, economic, and a health‐related variables between the study areas. Another possible reason might be the poor food security and diversity in the study area, which are directly related to the undernutrition of the children.

Underweight, stunting, and wasting were found in 29.9%, 21.6%, and 10.2% of the children, respectively, which was higher than a study conducted in Northwest Ethiopia.^47^ The differences might be related to the direct causes of undernutrition, including the level of inadequate nutrition, recurrent infections, and chronic diseases that cause poor nutrient intake, absorption, or utilization of foods. Stunting is one indicator of undernutrition that causes a significant public health problems among SAC in Ethiopia, which may affect their physical and/or mental development. There was a high prevalence of stunting in the study area, whichis why Ethiopia, lounched Seqota declaration by the name of this study area that it would abolish child malnutrition by 2030.^18^

Dietary diversity score, meal frequency, lack of access to school‐based feeding, and parasitic infection were independently predicted undernutrition among SAC in this study. A similar association was reported between the dietary diversity score in Burkina Faso^49^ and lack of access to school‐based feeding in Mecha, Northwest Ethiopia,^47^ showed a statistically significant association. Furthermore, meal frequency and being infected with intestinal parasites were predictors of undernutrition, which is in agreement with a study conducted in Bahir Dar, Ethiopia.^19^ Intestinal parasitic infection competes for the nutritional intake of children and also impairs the immune system of the host, so that it makes them susceptible to many diseases.

### 4.1 Limitation of the study

The limitation of the study was that we did not measure the intensity of the parasites, micronutrient intake, and red blood cell levels of the children due to a lack of laboratory equipment and reagents. As a result, we recommend that future studies of this type take into account the limitations mentioned above.

## 5 CONCLUSION AND RECOMMENDATIONS

The prevalence of both intestinal parasitic infections and undernutrition was high among SAC in Sekota Town. Intestinal parasitic infection was significantly associated with the drinking water source, the habit of open‐field defecation, undernutrition. Dietary diversity score, meal frequency, lack of access to school‐based feeding, and being infected with intestinal parasites were independent predictors of undernutrition among SAC in this study. The results imply the need for strengthening integrated strategies for the reduction of intestinal parasitic infections and undernutrition. The findings of this study will be an important input for the federal government or local government in modifing the intestinal parasite prevention and control programs and Seqota Declaration initiatives as well.

## AUTHOR CONTRIBUTIONS

**Habtu Debash**: Conceptualization; data curation; formal analysis; funding acquisition; investigation; project administration; resources; software; supervision; validation; visualization; writing–original draft; writing–review and editing. **Megbaru Alemu**: Conceptualization; data curation; formal analysis; funding acquisition; investigation; methodology; resources; software; supervision; validation; visualization; writing–review and editing. **Habtye Bisetegn**: Conceptualization; data curation; formal analysis; funding acquisition; investigation; methodology; project administration; resources; software; supervision; validation; visualization; writing–review and editing.

## CONFLICT OF INTEREST STATEMENT

The authors declare no conflict of interest.

## ETHICS STATEMENT

Consent for publication is not applicable as individual data such as images and videos did not accompany this manuscript.

## TRANSPARENCY STATEMENT

The lead author Habtu Debash affirms that this manuscript is an honest, accurate, and transparent account of the study being reported; that no important aspects of the study have been omitted; and that any discrepancies from the study as planned (and, if relevant, registered) have been explained.

## Data Availability

All authors have read and approved the final version of the manuscript had full access to all of the data in this study and takes complete responsibility for the integrity of the data and the accuracy of the data analysis.
